# Medawar’s PostEra: Galectins Emerged as Key Players During Fetal-Maternal Glycoimmune Adaptation

**DOI:** 10.3389/fimmu.2021.784473

**Published:** 2021-12-15

**Authors:** Ellen Menkhorst, Nandor Gabor Than, Udo Jeschke, Gabriela Barrientos, Laszlo Szereday, Gabriela Dveksler, Sandra M. Blois

**Affiliations:** ^1^ Department of Obstetrics and Gynaecology, University of Melbourne, Melbourne, VIC, Australia; ^2^ Gynaecological Research Centre, The Women’s Hospital, Melbourne, VIC, Australia; ^3^ Systems Biology of Reproduction Research Group, Institute of Enyzmology, Research Centre for Natural Sciences, Budapest, Hungary; ^4^ Department of Obstetrics and Gynecology, University Hospital Augsburg, Augsburg, Germany; ^5^ Laboratorio de Medicina Experimental, Hospital Alemán—Consejo Nacional de Investigaciones Científicas y Técnicas, Ciudad Autónoma de Buenos Aires, Argentina; ^6^ Medical School, Department of Medical Microbiology and Immunology, University of Pecs, Pecs, Hungary; ^7^ Department of Pathology, Uniformed Services University, Bethesda, MD, United States; ^8^ Department of Obstetrics and Fetal Medicine, University Medical Center Hamburg-Eppendorf, Hamburg, Germany

**Keywords:** galectins, pregnancy, pregnancy specific proteins, glycoimmunology, placenta

## Abstract

Lectin-glycan interactions, in particular those mediated by the galectin family, regulate many processes required for a successful pregnancy. Over the past decades, increasing evidence gathered from *in vitro* and *in vivo* experiments indicate that members of the galectin family specifically bind to both intracellular and membrane bound carbohydrate ligands regulating angiogenesis, immune-cell adaptations required to tolerate the fetal semi-allograft and mammalian embryogenesis. Therefore, galectins play important roles in fetal development and placentation contributing to maternal and fetal health. This review discusses the expression and role of galectins during the course of pregnancy, with an emphasis on maternal immune adaptions and galectin-glycan interactions uncovered in the recent years. In addition, we summarize the galectin fingerprints associated with pathological gestation with particular focus on preeclampsia.

## Overview: Galectin-Glycan Interactions at the Maternal Immune Function

Almost fifty years ago (mid-1970s), galectins were first described as a family of evolutionarily conserved animal ß-galactoside binding lectins (1). Since then, the importance of galectins has been recognized in many biomedicine disciplines including inflammation, malignancy and reproductive biology. Galectins have extensive roles in the regulation of cell differentiation and function, but it is their ability to control the innate and adaptive immune system in healthy and disease states which has attracted significant interest. Galectins are well established regulators of lymphocytes, especially T lymphocyte development, differentiation, activation and effector function (2). Multiple members of the galectin family are widely expressed at the maternal-fetal interface where they play important roles in implantation, placental and fetal development, including in the regulation of maternal-fetal tolerance. Here, we review uterine and placental galectin expression, function, and glycan interactions at the maternal-fetal interface and highlight placental pathologies associated with an aberrant galectin signature.

## Galectins: Structure and Function

**Figure 1:**
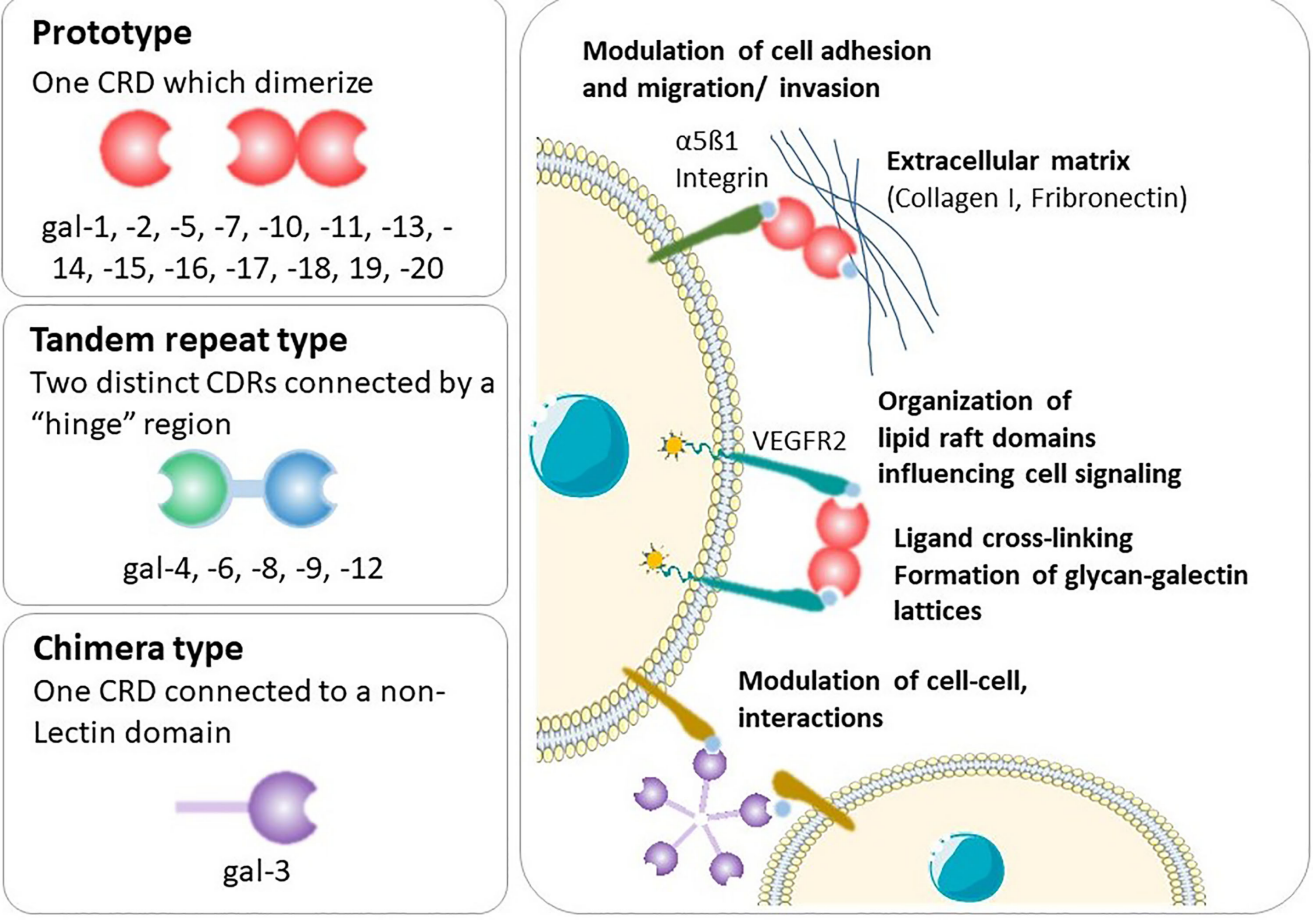
Types of galectins and general mechanisms. Shown is an illustration of the galectins structure and the functional interactions of this type of lectins with cell-surface and extracellular glycoconjugates.

Galectins are a highly evolutionarily conserved family, of which more than 20 galectins have been identified in mammals (3) and 12 in humans (4, 5). Galectins are found on many chromosomes including human chromosomes 1, 11, 14, 17, 19 and 22. A cluster of galectins found on chromosome 19 includes the placental-specific galectins (gal-13, -14, -16) (6), which are thought to be important in the formation of the highly invasive placenta found in humans. Galectins possess a characteristic carbohydrate recognition domain (CRD) and are classified according to their structure as prototype, chimera or tandem-repeat type (**Figure 1**) (7). Prototype galectins (gal-1, -2, -5, -7, -10, -11, -13, -14, -15 and -16) contain one CRD and typically form non-covalently linked homodimers (3). The only chimera-type galectin (gal-3) consists of a C-terminal CRD connected to a N-terminal ‘tail’ that facilitates oligomerization into trimers and pentamers (8). Tandem-repeat type galectins (gal-4, -6, -8, -9, -12) contain two distinct CRDs connected by a linker peptide (2, 3).

Following synthesis in the cytoplasm, galectins are predominantly secreted into the extracellular space where they bind to carbohydrate ligands on the cell surface or in the extracellular matrix. Alternatively, they can be translocated to the nucleus, where they form part of the spliceosome (3). Galectins lack a classical secretion sequence; thus, galectin secretion is non-classical, bypassing the Golgi complex, likely effected by direct transport across the plasma membrane (3). Whilst the N-acetyllactosamine-enriched glycoconjugates binding capacity of galectins led to their discovery (9), galectins generally only weakly bind to β-galactoside-containing glycans (7). Due to the unique nature of their CRDs, galectins selectively bind to specific ligands, but this binding is complex, regulated in part by the physiological concentration of the lectin and multivalency and oligomeric state of the galectin and ligand (7). Indeed, the physiological ligands of many galectins are unknown. Altogether, this means that the functions of galectins are highly contextual and reflect a dynamic mechanism to regulate cell function (2).

## Galectin-1

The first member of the galectin family to be identified, gal-1, is a homodimeric protein that belongs to the prototype subgroup and is composed of two subunits of 14.5 kDa (10). Gal-1 is synthesized on cytosolic ribosomes and translocated to the extracellular compartment through a non-classical secretory pathway. Recently, it is been described that upon sensing cytosolic lipopolysaccharide (LPS), gal-1 is released from the cell by caspase-11 associated mechanisms, a process which is dependent on gasdermin D (11). Thus, gal-1 acts as a danger-associated molecules pattern (DAMP) in the extracellular space to enhance the inflammatory response elicited by intracellular LPS. However, the gal-1 secretion pathway in a physiological situation such as pregnancy and placentation has not been determined. Intracellularly, gal-1 is predominantly monomeric and participates in protein-protein interactions in a carbohydrate-independent manner (e.g. with H-Ras (12), protocadherin-24 (13) and gemin4 (14) among others). Once in the extracellular compartment, gal-1 spontaneously dimerizes and binds to numerous glycoproteins mostly in a glycan-dependent manner (15). Thus, gal-1 recognizes galactose-β1-4–N-acetyl-glucosamine [N-acetyl-lactosamine (LacNAc)] units present on the branches of *N-* or *O-*linked glycans on diverse cell surface receptors (e.g. CD45 on lymphocytes) and extracellular matrix (ECM) proteins including laminin and fibronectin (16–18) (**Figure 1**). Gal-1 displays broad anti-inflammatory properties and targets multiples immune cell types. This review focuses on gal-1’s significance within the maternal immune system during pregnancy and its influence on pregnancy outcome.

At the feto-maternal interface, a wide range of immune and non-immune cells synthetize and secrete gal-1 (3). Gal-1 was shown to be the most abundantly expressed in the endometrium –when compared to all other human tissues (19), where it is mainly localized to decidual stromal cells. Gal-1 expression is sex hormone-dependent, therefore its expression is increased in the late secretory phase endometrium and it is further elevated in the decidua (20). *In vivo* evidence also confirmed that sex hormones (estrogens and progesterone) regulate the expression of uterine gal-1, which is strongly increased during embryo implantation (21) and then sustained through pregnancy. Interestingly, in the course of the emergence of placental mammals, conserved *cis* motifs were gained including an estrogen responsive element in the 5′ promoter of *LGALS1* (gal-1 gene) accounting for this sex steroid regulation of *LGALS1* expression (19). Of importance, gal-1 is one of the most strongly expressed proteins in the decidua, mainly in decidual stromal cells, at term gestation, with decreased expression in laboring women (22). Taken together with its anti-inflammatory actions in decidual cells, measured by its inhibition of LPS-induced IL-6 production in decidua-derived mesenchymal cells (23), gal-1 may regulate decidual immune cell populations and sustain a local anti-inflammatory microenvironment that favors pregnancy maintenance, and its decreasing expression at term may allow the pro-inflammatory changes in the decidua needed for the onset of labor.

Besides maternal decidual stromal cells, another major source of gal-1 are the fetal trophoblasts as observed in various species. Early in mouse and human embryogenesis, gal-1 is detected starting in the fourth/five day of development and its expression is limited to the inner cells mass and outer cells (which includes the trophectoderm) (24, 25). The trophectoderm of the blastocyst exhibits highly differentiated functions and participates in a complex dialogue with maternal cells that enables implantation. Indeed, gal-1 expression is strong in the trophectoderm-derived giant cells (GC) and in spongiotrophoblast subsets, layers of the placenta that are involved in placental invasion and have endocrine functions (26, 27). Despite differences between mouse and human placentation, gal-1 expression is also prominent in the human trophoblast populations that carry out interstitial and endovascular invasion, and regulate the ability of extravillous trophoblasts to secrete immunoregulatory proteins such as HLA-G (25, 26, 28–30). Moreover, early-gestation chorionic villus–derived placenta mesenchymal stromal cells express prominent gal-1 on the surface and this lectin is abundant in secreted exosomes, which has been recently suggested to have neuroprotective effect (31). This opens up new avenues in the field of gal-1 as critical signaling molecule in the placenta secretome.

Decidual NK (dNK) cells are one of the best examples of how maternal immune-privileged sites shape the phenotype of leukocytes (32). dNK cells have increased gal-1 expression when compared to peripheral NK cells (33, 34). Toscano M et al. showed that gal-1 selectively controls the fate of Th1 and Th17 cells, due to the glycan-repertoire expressed by these T cells that is compatible with gal-1 binding, whereas Th2 cells are resistant to this lectin as a result of increased α2,6-sialylation of their cell surface glycoproteins (35). Based on their ability to secrete gal-1, dNK cells have been proposed to induce apoptosis of activated Th1/Th17 cells (33). It is not clear, however, whether the gal-1 expressed on dNK cells is also present in cytotoxic granulates as has been recently showed in CD8^+^ cytotoxic T lymphocytes (CTLs) (36). Interestingly, under inflammatory conditions such as during the course of preeclampsia, peripheral NK cell-subsets depicted a less prominent gal-1 expression, which may be responsible for the exacerbated Th1/Th17 systemic response (37). Although dNK cells generally have a less inflammatory phenotype compared to their peripheral counterparts, there is evidence for anti-viral activity of dNK cells (38).

With regards to antigen presenting cells in the decidua (e.g. dendritic cells, DCs), we found that exogenous gal-1 fine tunes the DC immunoregulatory properties (39). Specifically, gal-1 treated decidua DCs induced an IL-10 expressing Treg cell subset that is compatible with healthy gestation (39). In contrast, during a failing pregnancy, an uncontrolled Th1 response is accompanied by an immunogenic DC phenotype and decreased decidual gal-1 expression (39, 40). Although several gal-1 cell surface receptors including CD45, CD43, CD69, the pre-BCR, and vascular endothelial factor receptor 2 have been characterized, further studies are needed to identify the gal-1 receptor(s) at the feto-maternal interface responsible for the extracellular function of this lectin. In addition, characterization of the glycosylation profile of the potential decidual gal-1 ligands, will provide their functional relevance in the gal-1-glycan axis that fine tunes the immune response during gestation. Mast cells are found in mucosal and epithelial tissues including the feto-maternal interface and these cells secrete gal-1 during the course of pregnancy. Although mast cells are a small population, these cells are known to regulate vasodilation, vascular homeostasis, and angiogenesis. Therefore, the expression of gal-1 in this particular cell subtype can be related to increased angiogenesis and may indirectly regulate placentation (41).

Given the important immunomodulatory and anti-inflammatory roles of gal-1, it is unsurprising that dysregulated gal-1 production is found in complications of pregnancy associated with impaired immune tolerance including early pregnancy loss (42–44). Specifically, placental gal-1 expression and maternal blood gal-1 levels are downregulated in the first trimester in women who miscarry (25). In preeclampsia, another disease associated with inflammation, decreased maternal blood levels of gal-1 are found in the second trimester, while its increased expression in the placenta and elevated maternal blood concentrations can be detected at the time of clinical diagnosis (42–44). In murine models, loss of gal-1 causes preeclampsia-like features, exemplified by exacerbated inflammation and an anti-angiogenic maternal response, which is associated with reduced placental labyrinth area and impaired spiral artery remodeling (39, 45). Overall, these data suggest that a substantial decrease in gal-1 expression in early pregnancy may lead to the complete loss of tolerance and result in miscarriage in both species, while a lower level of inhibition of gal-1 expression at the feto-maternal interface, may enable the further progression of pregnancy but with disturbed maternal-fetal immune interactions, leading to preeclampsia and chronic rejection of the fetal semi-allograft. The observed discrepancy in gal-1 levels between the second and third trimesters in preeclampsia may reflect an initially inhibited expression of gal-1, which may result in a compensatory overexpression later in pregnancy similar to what is detected in rejected kidney allografts (46). Because preeclampsia and early and recurrent pregnancy loss (RPL) are syndromes rather than unique entities with diverse etiologies (47), is it important to emphasize that only a portion of the clinical cases exhibit the above immune/anti-angiogenic pathologies related to gal-1 dysregulation.

## Galectin-2

Galectin-2 (gal-2, encoded by the *LGALS2* locus) belongs to the prototype subtype and its function at the feto-maternal interface is less understood than that of other galectins (1, 48). This lectin has been linked with pro- as well as anti-inflammatory actions (49, 50). The cellular binding sites for gal-2 are β1 integrin on T cells or closely associated glycoproteins (51). Binding of gal-2 to leukocytes results in cell-specific responses including apoptosis of activated T cells and regulation of leukocyte turnover (52, 53). In mice, gal-2 suppresses contact allergy reactions by inducting the apoptosis of activated CD8^+^ T cells; however, it has no significant effect on resting CD8^+^ T cells (54). Additionally, gal-2 has been attributed important inhibitory functions in monocyte and macrophage physiology, acting to inhibit monocyte migration and preventing macrophage-induced T cell activation (55). Thus, gal-2 was shown to shift T-cell cytokine profiles towards a Th2 phenotype, which is accompanied by downregulation of interferon (INF)-γ, tumor necrosis factor (TNF)-α and upregulation of IL-5 (52). In activated neutrophils, gal-2 induced externalization of phosphatidylserine leading to phagocytosis (53). The therapeutic potential of gal-2 has been demonstrated in acute and chronic mouse colitis disease models, where gal-2 induced a reduction in inflammation (49).

In the normal first trimester placenta, gal-2 is strongly expressed in the syncytiotrophoblasts (STB) with its cellular localization mainly found at the sites of interaction with the maternal blood. However, gal-2 expression is not restricted to the STBs as nuclear gal-2 expression has also been described in decidual cells (56). Expression of gal-2 within the placenta is reduced in pregnancy complications including miscarriages, preeclampsia and intrauterine growth restriction (IUGR) pregnancy (56–59). Preeclampsia not only causes a dysregulation of the placental gal-2 expression, but Charkiewicz et al. have shown that alterations of gal-2 levels can also be detected in the maternal circulation (60). However, whether gal-2 expression is causal for the development of miscarriage, preeclampsia or IUGR or if it is a consequence of failed trophoblast invasion is yet to be elucidated (61). Interestingly, gal-2 expression during normal pregnancy varies with fetal sex. Male placentas show more prominent gal-2 expression in the extravillous trophoblast compartment when compared to the age-matched female placentas (58). Thus, only male placentas suffering from IUGR showed a reduced gal-2 expression in this trophoblast population, whilst female placentas remained unchanged. A gender-specific role for gal-2 in the aetiology of preeclampsia and IUGR should be considered and further investigated.

DNA methylation in the *LGALS2* gene may be an important mechanism to regulate gal-2 expression. It has been shown that maternal eating disorders affect offspring cord blood DNA methylation (62). In this context, offspring of women with active restrictive eating disorders in pregnancy had lower whole-genome methylation compared to offspring of women with past restrictive eating disorders (62). In addition, increased methylation at the *LGALS2* locus could be identified in offspring of women with past eating disorder compared to controls (62).

## Galectin-3

Galectin 3 (gal-3, encoded by the *LGALS3* locus) is the only chimera-type member of the family identified so far (8). *Via* its CRD, which is shaped as a cleft open at both ends, gal-3 exerts high affinity binding to poly LacNAc extensions of core 2 O- and complex N-glycans as well as to ABH blood group oligosaccharides (63).

Gal-3 is present both extracellularly and in various subcellular compartments including the cell membrane, nucleus and cytoplasm; where its unique chimeric structure allows it to interact with a variety of ligands to modulate specific processes including cell growth and survival, adhesion, migration, invasion, immune function and angiogenesis, all of which play a significant role during maternal adaptation to pregnancy. In the context of immune adaptations, a dual role in the regulation of apoptosis depending on its subcellular localization has been proposed for gal-3, promoting T cell apoptosis when secreted to the extracellular milieu (64, 65) and showing protective effects when acting intracellularly (66, 67). In addition, gal-3 has been shown to exert potent inhibitory effects on NK cell activation and function, by either binding specific core 2 O- glycan moieties on target cells (68, 69) or by directly interacting with NK cell activating receptors [i.e., NKp30, (70)]. Gal-3 is recognized as a potent modulator of innate and adaptive responses, being involved in the activation and differentiation of a variety of immune cell subsets. It can regulate several components of the acute inflammatory response including neutrophil activation and rolling (71, 72), chemoattraction of monocytes/macrophages (73) and mast cell degranulation (74). Among other mechanisms, gal-3 modulates adaptive immunity by enhancing tolerogenic (i.e., regulatory) IDO-expressing DC that support Treg expansion (75), acting extracellularly to inhibit TCR signaling at the immunological synapse (76) and by interfering with co-inhibitory receptor signaling [i.e., PD-1 and LAG-3, (77)]. Intracellularly, gal-3 appears to be critical for supporting OX-40 mediated development of memory CD8+ T cells following antigen exposure (78). Additionally, under sustained tissue damage, gal-3 can promote the transition to chronic inflammation, quenching T cell responses (64, 79) and facilitating the walling off of tissue injury with fibrogenesis and organ scarring (80). In damaged cells, the lectin can also function as a receptor for advanced glycation end products promoting accumulation of reactive oxygen species and endothelial dysfunction (81, 82), which are both considered a hallmark of preeclampsia pathogenesis. More recently, intracellular galectins including gal-3 have emerged as important mediators sensing tissue damage in the context of infections given their ability to recognize host glycans exposed to the cytosolic milieu upon rupture of endocytic vesicles or organelles (83), which in turn leads to activation of autophagy pathways and recruitment of antimicrobial factors. These functions, along with its proven ability to control immune responses through DAMP and (pathogen-associated molecular patterns) PAMP pathways (84, 85), make gal-3 an important mediator in both defense from microbial infections and development of autoimmune conditions. Determining whether these novel functions of gal-3 play a significant role during the resolution of infections at the maternal fetal interface represents an attractive subject for further research.

Despite considerable research over the past years, the functional implications of gal-3 in the context of pregnancy are only just emerging. In humans, gal-3 mRNA and protein can be detected in maternal decidual cells (86) and also in all trophoblast lineages of the placenta (87), with increased expression levels associated with differentiation of the cytotrophoblasts along the invasive extravillous trophoblast pathway (87, 88). In line with these findings, recent *in vitro* studies have identified gal-3 as part of the trophoblast invasion machinery (89) as well as a positive modulator of crucial trophoblast cell functions including capillary tube formation and syncytialization (88). In mice, gal-3 is detected primarily in the uterine luminal and glandular epithelium, where its timely up-regulation appears to be a requisite for successful implantation (90). A role for this lectin in driving proper placental function is further emphasized in a recent study, which demonstrated that *Lgals3* deficiency is associated with impaired differentiation of trophoblast layers, enhanced decidual NK cell infiltration and cytotoxic degranulation, and defective vascularization resulting in asymmetric growth restriction due to placental insufficiency (91). Interestingly, these studies revealed a differential contribution of gal-3 to placental function depending on its source of expression, as the IUGR phenotype was only reproduced in mating models with *Lgals3* deficiency of maternal origin. Furthermore, dysregulated placental expression of gal-3 associated with enhanced activation of cellular stress pathways was recently reported preceding the establishment of the maternal syndrome in an experimental model of preeclampsia superimposed on chronic hypertension (92).

During the course of pregnancy, maternal circulating gal-3 levels increase as pregnancy progresses (91). Dysregulation of systemic levels of gal-3 have been described in pregnancy complications, particularly the so-called ‘Great Obstetrical Syndromes’ including preeclampsia (42, 93), IUGR (91), preterm birth and premature rupture of membranes (94, 95), and spontaneous pregnancy loss (96), all of which are associated with disorders of deep placentation. However, an important limitation of these studies is the diversity of criteria used to establish patient cohorts, which leads to conflicting results that make it difficult to determine whether dysregulated gal-3 levels reflect a causative link or appear as consequence of the placental pathology. In this context, future studies evaluating placental biology, the regulation of vascular responses and maternal adaptations in experimental models with altered gal-3 expression represent exciting avenues of research to establish the precise physiological role of this lectin during pregnancy as well as its potential application in diagnosis and interventions in pathological settings.

## Galectin-7

Galectin-7 (gal-7) is a prototype galectin initially identified as a marker of stratified epithelia that is also expressed by many other types of epithelia and other cell types including lymphocytes (97). The *LGALS7* gene is found on chromosome 19 (98), in a cluster of galectins which includes gal-4 and the placental-specific galectins gal-13, -14 and -16. In humans there is a duplicate copy of *LGALS7* (*LGALS7B*) located adjacent to *LGALS7* but on the opposite strand of chromosome 19 (99). Gal-7 is able to form homodimers with a ‘back-to-back’ organization and can be secreted despite having no cell secretion motif (97, 98). Gal-7 acts intracellularly *via* interactions with c-Jun N-terminal kinases, Ras or Bcl-2 and extracellularly *via* paracrine mechanisms to induce gene transcription, including autocrine amplification (100, 101 *,* 102–106).

Gal-7 production is regulated by p53, TNF-α and NFĸB (97), but based on upstream promotor region sequencing it is predicted that *LGALS7* and *LGALS7B* may each be regulated by different transcription factors (97, 99, 107). Studies to date suggest that gal-7 has key functions in epithelial cell homeostasis, including cell growth, differentiation and apoptosis as well as functions in cell adhesion, migration and immune cell regulation (101, 108 *;* 97, 105, 106, 109–116). The specific function of gal-7 likely relates to its cellular localization as gal-7 has been detected in the nuclear, cytosolic, mitochondrial and extracellular compartments (117).

Gal-7 has been suggested to play a key role in the intracellular immune response against infection. Gal-7 is recruited by Tollip to the autophagosome in HaCaT cells undergoing bacterial autophagy following group A *Streptococcus* infection (118). Gal-7 may also be considered an alarmin as it is released from human epithelial keratinocytes *via* an IL-4/IL-13/STAT6 dependent mechanism, it polarizes CD4+ T cells towards a Th1 phenotype and was found to be overexpressed in a murine model of cardiac allograft rejection (111, 115, 119).

*In vivo* administration of exogenous gal-7 to pregnant mice during mid-gestation results in a pro-inflammatory response with elevated placental IL-1*β* and IL-6 and reduced IL-10 mRNA production (120). Other reports however, indicate that gal-7 has anti-inflammatory actions treatment of PMA-stimulated Jurkat cells with gal-7 inhibits IL-2 and INF-γ production (112), gal-7 silencing elevates IL-17A-stimulated HaCaT cell secretion of IL-6 and IL-18 (107) and gal-7 is down-regulated in a mouse model of psoriasis (107). Therefore, the immune regulatory role of gal-7 may be cell-type specific and/or may depend on its cellular localization.

The absence of detectable gal-7 mRNA in term placenta (6) and the discovery that *Lgals7* deficient mice are fertile and give rise to normal and fertile offspring initially suggested that gal-7 might not play an important role in female reproduction (121). More recently, immunohistochemical studies localized gal-7 to endometrial luminal and glandular epithelial cells, menstrual phase stromal cells and 1^st^ trimester and term placental trophoblast including STB, cytotrophoblasts and extravillous trophoblasts (113, 114, 122, 123). The published reports also suggest that gal-7 localizes to uterine/decidua-resident immune cells however, the identity of the immune cells was not confirmed by dual staining (114, 122).

Exogenous gal-7 promotes endometrial epithelial cell adhesion and migration *in vitro* (113, 114). Gal-7 is present in menstrual fluid and *in vitro* studies suggest that it enhances re-epithelialization during endometrial repair after menstruation by promoting epithelial cell migration in a fibronectin/integrin dependent manner (113). Exogenous gal-7 enhances adhesion between human endometrial epithelial cells and the HTR-8/SVneo trophoblast cell line, suggesting that gal-7 may facilitate blastocyst adhesion to the uterine luminal epithelium during implantation. However, as gal-7 production is not up-regulated during the receptive phase of the menstrual cycle, it is unlikely that gal-7 is a critical mediator of blastocyst adhesion (114). Rather, somewhat paradoxically, endometrial epithelial production of gal-7 is increased in women with a history of multiple early pregnancy losses (114). Gal-7 is also abnormally elevated in maternal serum at 6 weeks gestation from women who subsequently miscarry but is not altered after week 7 of gestation (114, 124). The impact of even slightly increased gal-7 during early pregnancy may be substantial and sustained as it exhibits high stability due to the its protease resistance and increased stability following ligand binding (125). Elevated endometrial gal-7 may alter endometrial function leading to early pregnancy loss by increasing blastocyst-luminal epithelial adhesion allowing lower quality embryos to implant and altering the endometrial inflammatory environment by polarizing T cells towards a Th1 phenotype (114, 126–130).

Increased gal-7 production is found in early pregnancy chorionic villous samples (CVS; collected at 11-14 weeks gestation) from women who subsequently develop preterm preeclampsia (<37 weeks gestation) compared to women with uncomplicated pregnancies (120). A small retrospective cohort study also identified abnormally elevated gal-7 in maternal serum (collected at 10-20 weeks gestation) from women who subsequently developed preeclampsia (122). *In vivo*, gal-7 administration to pregnant mice causes preeclampsia-like features including hypertension, albuminuria and impaired placentation (120). Non-pregnant mice treated with gal-7 do not develop hypertension or albuminuria, demonstrating that gal-7 acts *via* the placenta to cause preeclampsia-like features in this model. Gal-7 regulates placental expression of many pathways thought to underlie the etiology of preeclampsia, including stimulating human placental villous production of *sFlt-1-e15a*, the sFlt-1 splice variant present only in the placenta of higher-order primates (120, 131). In mice, exogenous gal-7 induces a pro-inflammatory placental state (elevated *IL-1β*, *IL-6* and reduced *IL-10* mRNA) and alterations in the circulating and tissue renin-angiotensin-(aldosterone)-system (RAS) homeostasis (120, 132–137). To our knowledge this is the only mouse model of preeclampsia which causes alterations to RAS homeostasis without interventions to silence/overexpress specific RAS factors or surgically reducing uteroplacental perfusion and therefore could be a useful model to understand the role of RAS in the etiology of preeclampsia. Murine and *ex vivo* primary human trophoblast outgrowth experiments demonstrate that elevated placental gal-7 impairs trophoblast invasion (120) as is reported in preeclampsia (138). Gal-7’s inhibition of trophoblast invasion may occur *via* regulation of Pappalysin-2 and Disintegrin and metalloproteinase domain-containing protein 12 (ADAM12) (120) which are well established regulators of trophoblast migration (139–141).

Altogether these studies suggest that gal-7 likely has a role in the uterine and placental innate immune response and that it is produced at low levels during uncomplicated pregnancies. Elevated production of gal-7 during any stage of pregnancy may reflect or induce a pro-inflammatory state leading to poor pregnancy outcomes including RPL and preeclampsia.

## Galectin-9

This “tandem-repeat type” galectin encoded by the *LGALS9* gene located on human chromosome 17 is a 34-39 kDa protein (142). Gal-9 undergoes post-transcriptional splicing to form many splice variants. Among tandem-repeat galectins, gal-9 has a wide expression pattern including the nuclear, cytoplasmic and extracellular compartments (143, 144). The exact mechanism of secretion for gal-9 is poorly understood, however, it is most likely to be secreted by one of the following non-classical secretory pathways: direct translocation across the plasma membrane, release *via* packaging in exosomes, or export *via* lysosomes, endosomes, and microvesicles (145, 146).

Among several identified gal-9 receptors, T-cell immunoglobulin mucin 3 (Tim-3) is the most extensively studied. TIM-3 was first discovered in 2002 on interferon IFN-γ producing CD4^+^ (Th1) and CD8^+^ T cytotoxic cells (147). TIM-3 expression was verified in various immune cells, including Th1, Th17, NK cells, NKT-like cells, γ/δ T cells, Tregs, MAIT-like cells, dendritic cells, monocytes and also on trophoblast such as giant cells (148–151). A growing body of evidence supports a role of Tim-3 signaling in shaping both the adaptive and innate immune responses (152). There is evidence that engagement of Tim-3 by its ligand gal-9 leads to the death of Th1 and Th17 cells, influencing the ability to induce T cell tolerance in both mice and humans (153–156). CD4^+^ T cells secrete gal-9 upon T-cell receptor activation resulting in the regulation of Th17/Treg development (157). Gal-9 suppresses Th17 cell differentiation and induces the apoptosis of Th1 and cytotoxic T cells while in mice, it has been shown to enhance regulatory T cell differentiation, suggesting immunosuppressive functions (158). Moreover IL-6 abrogates the increase of gal-9^+^ Th cells *in vitro* and indicates that the neutralization of IL-6 may be a strategy to increase gal-9^+^ Th cells to ameliorate Th1/Th17-skewed immunity (157). Thus, engagement of TIM-3 by gal-9 may function as a negative regulator, abrogating Th1- and Th17-driven immune responses and may modulate the Th1/Th2 balance. The outcome of this interaction could have essential roles in human pregnancy.

Gal-9 is highly expressed in the female reproductive tract and at the maternal-fetal interface (86, 159) (160). In mouse models, gal-9 plays a role in cell-to-cell interactions and the establishment of an immune-privileged local environment for implantation and early fetal development, as well as the mediation of decidual cell migration and chemotaxis (159). Both murine placental spongiotrophoblast and decidual regulatory T cells express gal-9: decidual gal-9^+^ Th cells are an important source of a secreted, soluble form of gal-9 (161). Endometrial stromal cells secrete gal-9 to suppress inflammatory reactions of uterine NK cells *via* Tim-3 [23]. The predominant gal-9 splice variant (*Lgals9 D5*) was found to downregulate IFN-γ production mouse dNK cells and therefore gal-9 may limit Th1 cell numbers (158). Available evidence also supports a similar role for gal-9 in human pregnancy. Gal-9 is expressed by human endometrial glandular epithelial cells (uterodomes) during the window-of-implantation. Post-implantation, gal-9 is highly produced by epithelial cells and stromal cells of the decidua during early pregnancy and by syncytiotrophoblast and cytotrophoblasts (160, 162, 163). Gal-9 is implicated in the regulation of dNK cell function and the maintenance of normal pregnancy as the expression of gal-9 by primary human trophoblasts induces the transformation of peripheral NK cells into dNK-like cells *via* the interaction with Tim-3 molecule (164). Treg cells increase their gal-9 expression as pregnancy progressed-coinciding with the increasing gal-9 level in maternal blood, suggesting that expression of gal-9 in the Tregs subset may have important roles in the maintenance of pregnancy (165).

The evidence discussed above suggests that gal-9 is implicated in the modulation of maternal immune tolerance to support fetal growth and development, therefore it is important to consider its role in pathologic pregnancies like spontaneous miscarriage, RPL, or preeclampsia. Normal pregnancy and cases of spontaneous abortions differ significantly in terms of endometrial gal-9 splice variant profiles in both a mouse model of spontaneous abortion (CBA/J females with DBA/2J males) and in humans (158). Placentas from abortion-prone mice had lower mRNA levels of gal-9 compared to the normal placenta (158). Tim-3 expression by dNK cells from human miscarriages and abortion-prone mouse models is also reduced compared to healthy pregnancies, and the function of Tim-3^+^ dNK cells has been shown to be impaired (164). Moreover, decreased Th2 cytokine and increased Th1 cytokine levels were observed in Tim-3^+^ dNK cells, but not in those Tim-3^-^ dNK cells derived from human and murine miscarriages (164). These data indicate that both the frequency and the function of Tim-3^+^ dNK cells are abnormal in miscarriage. In addition, women with a history of RPL have lower levels of circulating gal-9 compared to healthy pregnant women and reduced Tim-3 expression by peripheral NK cells (166, 167). The dysregulation on the gal-9/Tim-3 axis is accompanied by a decrease of systemic TGF-β1 levels, which has been implicated in the upregulation of Tim-3 expression (162, 168). Besides these changes, an increase of soluble Tim-3 (sTim-3) in addition to the reduced gal-9 circulating levels has been reported in these patients, which could enhance the competitive binding of gal-9 by sTim-3 leading to failed inhibitory signals controlling inflammation (166). A therapeutic potential of recombinant (r)gal-9 has been shown in preclinical models of several diseases like transplant rejections and autoimmune condition (169). The use of rgal-9 in RPL patients might be an effective therapeutic strategy to restore maternal-fetal immune tolerance.

The gal-9/Tim-3 pathway has also been implicated in maternal systemic inflammation in early-onset preeclampsia, however further analyses are urgently needed for a better understanding of this axis (170, 171). Decreased Tim-3 expression by T cells, cytotoxic T cells, γ/δ T cells, NK cells, and CD56dim NK cells, as well as increased frequency of gal-9^+^ peripheral lymphocytes is detected in women with early-onset preeclampsia (148, 170). Hao et al. found that the gal-9 expression detected by immunofluorescence in decidual tissues from preeclamptic patients was significantly higher compared to the control group (171). These data suggest that the impairment of the gal-9/Tim-3 pathway can result in an enhanced systemic inflammatory response, including the activation of Th1 lymphocytes in early-onset preeclampsia. Furthermore, Li et al. demonstrated that exogenous rgal-9 administration alleviates the preeclampsia-like manifestations (characterized by insufficient trophoblast cell invasion and impaired spiral artery remodeling) in a rat model of preeclampsia induced by LPS by upregulating Tim-3 expression in decidual macrophages (172). This finding may be related to the activation of the gal-9/Tim-3 signaling pathway, which promotes decidual macrophage polarization shifting to M2 subtype (172). Thus, the gal-9/Tim-3 axis may provide a valuable target for clinical interventional immunotherapy for early-onset preeclampsia although the findings that the interaction of gal-9 with Tim-3 in innate immune cells such as monocytes and dendritic cells induces the secretion of pro-inflammatory cytokines including TNF-α and can synergize with Toll-like receptors should be taken into consideration (173). Further functional evidence is needed to better understand the *in vivo* extracellular functions of gal-9.

## Primate-Specific Placental Galectins (Gal-13, -14 and -16)

Gal-13 was discovered by Bohn et al. as Placental Protein 13 (PP13) when systematically isolating >50 pure proteins from the human placenta and characterizing them with physico-chemical methods (174). Using the purified PP13 protein and anti-PP13 antisera, Than et al. isolated and sequence analyzed the full-length cDNA encoding PP13, and described PP13 to be placenta-specifically expressed (175). They also discovered close placenta-specific galectin relatives of gal-13, and first characterized their evolution, structure, expression and functions (6, 176–182). Other groups, pioneered by Meiri et al. explored the clinical utilization of gal-13/PP13 immunoassays in maternal blood in normal and complicated pregnancies (183–186), as well as the potential use of gal-13/PP13 therapy for preeclampsia (187–193). As a result of systematic work, almost four decades of increasing international collaboration delineated the pivotal role of placental galectins in maternal-fetal immune-interactions and their promising diagnostic and therapeutic potentials for pregnancy complications.

The gene encoding gal-13 (*LGALS13*) is located on chromosome 19 within a cluster of galectin genes and pseudogenes, which emerged *via* birth-end-death evolution in anthropoid primates. Evolutionary analysis suggested that this cluster were originated from an ancestral mono-CRD galectin and underwent multiple gene duplications, rearrangements and losses as well as sequential divergence and subfunctionalization (4, 6). In humans, five genes belong to this galectin cluster, out of which three (*LGALS13*, *LGALS14*, *LGALS16*) are uniquely expressed by the placenta (4, 6). RNA and protein level evidences demonstrated predominant expression of these galectins in the syncytiotrophoblast but not in the underlying progenitor cytotrophoblast, with lower expression in extravillous trophoblasts, amnion epithelium and fetal endothelium (6, 177, 178, 181). The expression of these galectin genes is developmentally regulated during villous trophoblast differentiation and syncytialization, with the strongest expression observed for *LGALS14* and *LGALS13* due to promoter evolution (181). The insertion of a primate-specific transposable element into the upstream region of an ancestral galectin gene introduced several binding sites for transcription factors fundamental for placental gene expression, leading to the gain of placental expression of these galectins. Further promoter changes *via* duplication or insertion of transposable elements led to varying placental expression levels of these galectin genes (181). For gal-13, it is released from the syncytiotrophoblast into the maternal circulation by secretion or shedding of microparticles (178, 179, 183, 194, 195). Due to their similar structural and expressional characters, we may suppose that gal-14 and gal-16 behave similarly to gal-13 regarding their placenta-maternal transfer.

The placenta-specific galectins structurally belong to the “proto-type” sub-group of galectins, which have a single CRD (6, 176, 177, 196). The topologies of these CRDs revealed by homology modelling are similar to other prototype galectins, often called as “jelly-roll” structure, although their amino acid sequence has considerably diverged during evolution (4, 6, 176, 197). In fact, adaptive evolution occurred in the CRDs of newly emerged genes in the *LGALS13* clade, followed by conservation of residues in descendant lineages, suggesting that placenta-specific galectins have acquired and sustained important novel functions in anthropoids (4, 6). From the eight conserved residues in the CRD, three that are key for overall sugar binding were subject to strong purifying selection, while five residues crucial for the binding of galactose or glucose moieties were replaced in several lineages following gene duplications, which have contributed to differences in their CRDs (6). In accordance with this evolutionary evidence, *in vitro* sugar-binding assays and *in silico* ligand docking simulations showed that placenta-specific galectins have sugar-binding capabilities different from other galectins (6, 176, 177, 180), which was recently also supported by X-ray crystallographic studies (198–201).

The first *in vitro* functional assays with recombinant placenta-specific galectins demonstrated their pro-apoptotic capability on activated T lymphocytes in a similar extent as gal-1, thus, it was proposed that these galectins emerged to reduce the danger of maternal immune attacks on the fetal semi-allograft and to confer additional immune tolerance mechanisms in anthropoid primates, supporting their hemochorial placentation during long gestation (6, 197). Later, gal-13/gal-14 were shown to increase the rate of late-apoptotic T cells irrespective of their activation status (182). Since the surface expression of CD95 was also induced by gal-13/gal-14 treatment, these galectins may increase the sensitivity of activated T cells for activation-induced cell death (182). T cytotoxic cells bound more galectins and were more susceptible to gal-13/gal-14 induced apoptosis than T helper cells, probably due to their differential glycosylation patterns (182). An interesting observation was made on extracellular gal-13 aggregates localized around decidual veins in the first trimester (202). These crystal-like aggregates were associated with immune cell-containing zones of necrosis. It was hypothesized that syncytiotrophoblast-secreted gal-13 drains from the intervillous space into decidual veins, where it forms perivenous aggregates, which attract, activate and kill maternal immune cells while facilitating local tolerance for trophoblasts to invade and convert maternal spiral arterioles.

Unlike T cells, neutrophils cultured with recombinant gal-13 do not undergo apoptosis (203). Gal-13 did not interfere either with neutrophil extracellular trap (NET) release, degranulation, phagocytosis, or bacteria-induced reactive oxygen species (ROS) response, but induced increased expression of programmed death-ligand 1 (PD-L1), hepatocyte growth factor (HGF), vascular endothelial growth factor (VEGF), matrix metalloproteinase 9 (MMP-9), and TNFα (203). Thus, gal-13 may shift neutrophils towards a placental-growth-permissive phenotype, while maintaining all their primary functions and abilities to respond to bacterial invasion (203). In addition, gal-13/gal-14 stimulated IL-8 production in non-activated T cells (182), which is interesting in the context of the pro-angiogenic effect recombinant (204), decidual neutrophil-secreted or dNK cell secreted IL-8 (205–207). Since the pro-angiogenic effects of other galectins has been established as reviewed elsewhere (208), it is tempting to hypothesize that gal-13/gal-14 may induce angiogenesis at the maternal-fetal interface *via* the induction of T-cell secreted IL-8. Indeed, recent *in vivo* studies showed that exogenous gal-13 can induce 1) the vasodilatation of rat isolated uterine arteries by activating the endothelial prostaglandin and nitric oxide (NO) pathways (188, 189); 2) expansive remodeling of uterine veins and arteries (192); 3) drop in the blood pressure of rats (187, 188); and the 4) increase of placental and pup weights (188). These functional properties of placental galectins are remarkable especially in the context what we have learnt from the placental origins of pregnancy complications, especially preeclampsia, a multifactorial syndrome, in which disturbances in trophoblast-immune cell interactions and vascular remodeling are in the center of pathologies (209, 210).

Placental expression of gal-13 has been shown to be dysregulated before and after the preeclampsia onset, as evidenced at the RNA level in CVS first trimester samples, and remains at lower expression patterns by the time of disease diagnosis (178, 179, 181, 211, 212). A similar placental down-regulation was observed for gal-14 at the third trimester, and since its regulation of expression is strongly linked to that of gal-13 (181), we hypothesize that placental gal-14 behaves similarly to gal-13 in early pregnancy. Their down-regulation may lead to disturbed immune cell interactions and reduced trophoblast invasion as well as abnormal spiral artery remodeling and angiogenesis, leading to the ischemic stress of the placenta, which are central to the pathogenesis of preterm preeclampsia. In parallel with this, lower gal-13 concentrations are detected in the maternal circulation during the first trimester of women who subsequently developed preterm preeclampsia compared to healthy pregnant women by many international clinical studies, with some earliest ones referenced here (183–186, 197, 213–216). Due to its superior biomarker properties, gal-13 was assessed to be included into first trimester risk assessment of preterm preeclampsia by these and other studies. A seemingly conflicting observation was made, with preterm preeclamptic pregnancies showing a steep increase in maternal blood gal-13 concentrations during the second trimester, reaching significantly higher levels in the third trimester compared to healthy pregnancies (197). This phenomenon was elucidated to be due to the ischemic placental stress and the consequently augmented shedding of gal-13 from the placenta into the maternal circulation *via* trophoblastic microvesicles (178, 179, 195, 197, 217). Thus, placental galectins, especially gal-13, turned out to be promising early biomarkers of abnormal deep placentation. Moreover, encouraging studies are being performed for the replenishment of gal-13 to restore immune balance and inhibit the development of one of the most severe obstetrical syndrome, preeclampsia.

## Galectins and Pregnancy-Specific Glycoproteins

In addition to placental galectins, the fetus-derived syncytiotrophoblast, which is the major interface with the material circulation and represents the endocrine tissue of the placenta, secretes the pregnancy-specific glycoproteins (PSGs). PSG1 or Schwangerschafts protein 1 (SP1), as it was originally called, is one of 10 human PSGs and is increasingly secreted into the maternal circulation with advancing gestation. PSGs have been studied regarding their potential for being a biomarker for pregnancy complications and trophoblastic diseases for more than four decades although specific antibodies for the different PSGs are not available (218, 219). Recently, PSG expression was also observed in a subpopulation of extravillous trophoblasts at the mRNA and protein levels (220, 221). The presence of invasive trophoblast cells and the direct contact of maternal immune cells with fetal tissue as found in species with hemochorial placentas and in equine endometrial cups is hypothesized to have favored the evolution and expansion of PSGs, which exist only in a minority of mammals (222). The number of PSG genes varies greatly; while new world monkeys have 3 PSG genes, some old world monkeys have 17 (222). Humans have 10 PSG-encoding genes and splice variants that differ by the number of Ig-like domains or length of the cytoplasmic tail have been described (223). Interestingly, copy number variations are common in the human *PSG* locus and while some functions are shared by all members of the family, it remains to be determined whether during evolution, some members of the family have adopted new functions and bind to different ligands (224, 225). Therefore, whether higher PSG gene dosage and expression levels of individual PSGs confers an adaptive advantage or it is detrimental during pregnancy remains unknown (226).

Within the human PSGs, PSG1 is one of the highest expressed and the best studied (222, 227, 228). PSG1 has pro-angiogenic activity and immunomodulatory actions by binding to the latency-associated peptides of the anti-inflammatory cytokines TGF-β1 and TGF-β2 resulting in their activation and increase in the number of T regulatory cells (229–232). PSG1 also interacts with two integrins, α_5_β_1_ and α_IIb_β_3_, resulting in its ability to modulate extravillous trophoblast adhesion and migration and the interaction of platelets with fibrinogen, respectively (221, 227). We found that the concentration of PSG1 is lower in African American women diagnosed with preeclampsia when pregnant with a male fetus (233).

The high expression level of PSG1 during the third trimester permitted the isolation of native PSG1 from pooled serum of pregnant women using affinity chromatography allowing for a comprehensive glycomic and glycoproteomic investigation of the native protein (234). PSG1 has seven potential N-linked glycosylation sites across its four Ig-like domains designates as N, A1, A2 and B2 but only four sites mapping to the N, A1 and A2 domains of the protein were confirmed to be occupied by glycans (234). The presence of multiantennary and poly-N-acetyl-lactosamine (LacNac) elongated moieties with mainly alpha2,3-linked sialic acid terminals, suggested that PSG1 could interact with members of the galectin family. Because gal-1 has a well-established modulatory role in pregnancy-associated processes, the interaction between PSG1 and gal-1 has been investigated in detail (3, 39). Gal-1 binds to native and recombinant PSG1 in a carbohydrate-dependent manner, demonstrated by the inhibition of PSG1-gal-1 binding by lactose and failure of PSG1 generated in insect cells or N-acetylglucosaminlytransferase I (GnTI)-deficient cells, which carry only mannose-type glycans, to interact with gal-1. In addition, removal of N-linked oligosaccharides from the N- and A2- PSG1 domains by treatment with the amidase PNGase F prevented their binding to gal-1 (234). Changes in glycosylation could add further complexity to the regulation of important biological processes in pregnancy known to be regulated by both extracellular galectins and their ligands, including the PSGs. At present, whether glycosylation of PSGs and its ability to interact with galectins differs with gestational age or during pathological pregnancies has not been investigated. In addition, whether all members of the PSG family within a species or in the different species in which these proteins are expressed interact with gal-1 or other members of the galectin family has not been established and should be further explored.

## Conclusion and Perspective

**Figure 2:**
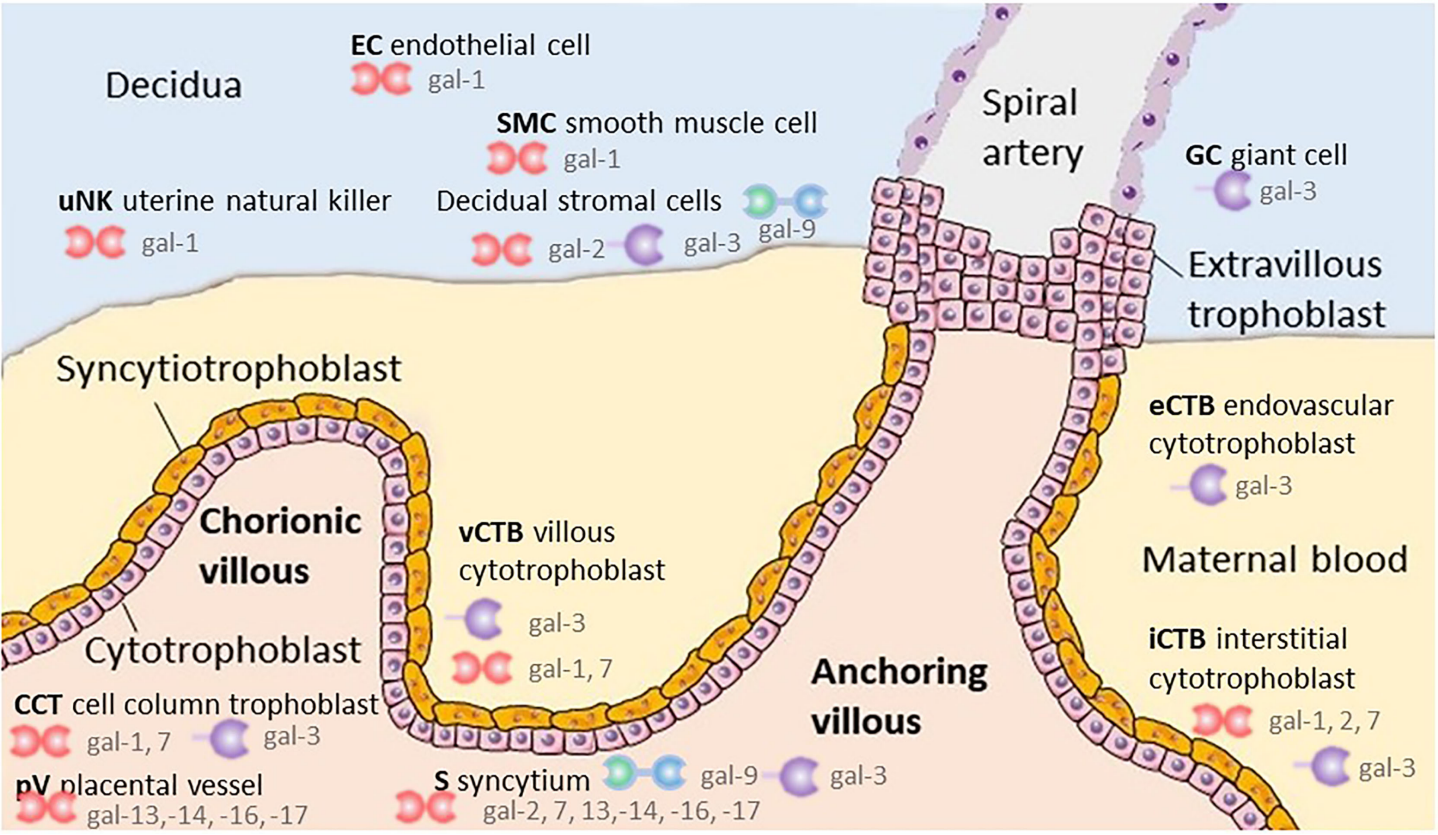
Localization of galectins within the placental and maternal compartment.

Galectins’ extracellular functions are mainly believed to be the result of their lectin properties, but intracellular functions, which are independent of their ability to bind glycans, result from protein-protein interactions (**Figure 2** and **Table 1**). The interaction of placental- or maternal- derived glycoproteins with galectins could potentially modulate the activity of some galectins once they are secreted from a cell. The final outcome of galectin-pregnancy specific glycoproteins interactions will depend on their concentration, that of other galectin ligands, and the affinity of the interactions. In addition, while only investigated for a few glycoproteins, the presence of specific glycan structures in proteins may vary at different times during gestation or under pathological pregnancy conditions. In this review, we have focused on the role of galectins in controlling the maternal immune adaptation to gestation; however, the galectins-glycans axis could modulate maternal immune response in the context of inflammation induced by microbes which requires further investigation. Deciphering those interactions will help to understand the critical role of glycoimmunology in the maternal adaptations and provide novel diagnostic and therapeutic targets for pregnancy complications characterized by aberrant glycosylation and imbalance of galectins.

**Table 1:** Galectins in the regulation of immune responses in pregnancy.

Galectin	Immunological role in pregnancy	Pathologies with aberrant expression	Proposed timing of initial insult	Evidence for causal role
Gal-1	Anti-inflammatory	↓ Early pregnancy loss	1^st^ trimester	Mouse model
		↓ Preeclampsia	1^st^ trimester	Mouse model
Gal-2	Anti-inflammatory	↓ Early pregnancy loss		
		↓ Preeclampsia		
		Intrauterine growth restriction (↓ Male placenta)		
Gal-3	Pro-inflammatory	↑ Early pregnancy loss	1^st^ trimester	
		↓ Intrauterine growth restriction	1^st^ trimester	Mouse model (maternal deficiency)
		↑ Preeclampsia	1^st^ trimester	Mouse model
		↑ Preterm birth and prelabor rupture of the membranes		
Gal-7	Pro-inflammatory	↑ Early pregnancy loss	Implantation	*In vitro* cell adhesion
		↑ Preeclampsia	1^st^ trimester	Mouse model; CVS
Gal-9	Anti-inflammatory	↓ Early pregnancy loss	1^st^ trimester	Mouse model
		↓ Preeclampsia	1^st^ trimester	Rat model
Gal-13	Anti-inflammatory	↓ Early pregnancy loss	1^st^ trimester	
		↓ Preeclampsia	1^st^ trimester	Rat model; CVS
Gal-14	Anti-inflammatory	↓ Early pregnancy loss	1^st^ trimester	
		↓ Preeclampsia	1^st^ trimester	

CVS, chorionic villous samples; ↓, down-regulated in pathology; ↑, up-regulated in pathology.

## Author Contributions

EM and SMB planned and wrote the review article, GD and NGT edited the manuscript. NGT (human gal-1 and placental galectins), UJ (gal-2), GB (gal-3), LS (gal-9), GD (PSG) contributed to the mentioned section. NGT and SMB drafted the manuscript figures. All authors contributed to the article and approved the submitted version.

## Funding

The writing of this review was supported by the Deutsche Forschungsgemeinschaft (BL1115/2-1, BL1115/4-1, Heisenberg program BL1115/7-1), and Heike Wolfgang Mühlbauer Stiftung to SMB. EM is supported by Mid-Career Fellowship, Department of Obstetrics and Gynecology, The University of Melbourne. NGT is supported by the Hungarian Academy of Sciences (Momentum LP2014-7/2014 grant) and the Hungarian National Research, Development, and Innovation Office (FIEK_16-1-2016-0005 and OTKA K124862 grants). GD is supported by the National Institutes of Health, USA (NIAID grant # R21AI156058).

## Conflict of Interest

The authors declare that the research was conducted in the absence of any commercial or financial relationships that could be construed as a potential conflict of interest.

## Publisher’s Note

All claims expressed in this article are solely those of the authors and do not necessarily represent those of their affiliated organizations, or those of the publisher, the editors and the reviewers. Any product that may be evaluated in this article, or claim that may be made by its manufacturer, is not guaranteed or endorsed by the publisher.
